# Differences in Dietary and Lifestyle Triggers between Non-Erosive Reflux Disease and Reflux Esophagitis—A Multicenter Cross-Sectional Survey in China

**DOI:** 10.3390/nu15153400

**Published:** 2023-07-31

**Authors:** Yang Chen, Xiaohong Sun, Wenjuan Fan, Jiao Yu, Peng Wang, Dong Liu, Mingwei Song, Shi Liu, Xiuli Zuo, Rong Zhang, Yuantao Hou, Shaomei Han, Yanqing Li, Jun Zhang, Xiaoqing Li, Meiyun Ke, Xiucai Fang

**Affiliations:** 1 Department of Gastroenterology, Peking Union Medical College Hospital, Chinese Academy of Medical Sciences and Peking Union Medical College, Beijing 100730, China; dr_chenyang@126.com (Y.C.); sunxiaoh2010@126.com (X.S.); songmingwei163@163.com (M.S.); houyuantao2013@163.com (Y.H.); lixiaoqing20060417@126.com (X.L.); kemypumch2006@aliyun.com (M.K.); 2 Department of Geriatrics, Peking Union Medical College Hospital, Chinese Academy of Medical Sciences and Peking Union Medical College, Beijing 100730, China; 3 Department of Gastroenterology, Tongji Hospital, Tongji Medical College, Huazhong University of Science and Technology, Wuhan 430030, China; juanwenfan1989@163.com; 4 Department of Gastroenterology, Union Hospital, Tongji Medical College, Huazhong University of Science and Technology, Wuhan 430022, China; liusu1982@163.com (J.Y.); 1986xh0568@hust.edu.cn (S.L.); 5 Department of Gastroenterology, Qilu Hospital of Shandong University, Jinan 250012, China; wangpeng_qd@163.com (P.W.); zuoxiuli@sdu.edu.cn (X.Z.); liyanqing@sdu.edu.cn (Y.L.); 6 Department of Gastroenterology, The Second Affiliated Hospital, Xi’an Jiaotong University, Xi’an 710003, China; liudong_doc@163.com (D.L.); zr4056@163.com (R.Z.); jun3z@163.com (J.Z.); 7 Department of Gastroenterology and Hepatology, Tianjin Medical University General Hospital, Tianjin 300052, China; 8 Department of Epidemiology and Statistics, Institute of Basic Medical Sciences, Chinese Academy of Medical Sciences & School of Basic Medicine, Peking Union Medical College, Beijing 100005, China; hansm5102@outlook.com

**Keywords:** gastroesophageal reflux disease, non-erosive reflux disease, reflux esophagitis, diet, lifestyle, triggers

## Abstract

The occurrence of gastroesophageal reflux disease (GERD) and symptom onset are closely associated with diet. We aimed to compare the dietary and lifestyle triggers between non-erosive reflux disease (NERD) and reflux esophagitis (RE) in Chinese patients and to provide evidence for development of practical dietary modifications for GERD. A multicenter cross-sectional survey was conducted. A total of 396 GERD patients with typical gastroesophageal reflux symptoms who received upper endoscopy in the previous month were enrolled, including 203 cases of NERD patients and 193 cases of RE patients. All participants completed questionnaires including demographic data, reflux symptoms, previous management, dietary and lifestyle habits, triggers of reflux symptoms, psychological status, and quality of life. There were no significant differences in GERD symptom scores between NERD and RE. RE patients had a higher male proportion and smoking/drinking and overeating rates than NERD patients. In the NERD group, more patients reported that fruits, dairy products, yogurt, bean products, cold food, and carbonated beverages sometimes and often induced reflux symptoms and had more triggers compared to RE patients. The number of triggers was positively correlated to GERD symptom score and GERD-HRQL score in both NERD and RE patients. However, 74.0% of GERD patients still often consumed the triggering foods, even those foods that sometimes and often induced their reflux symptoms, which might be related to the reflux relapse after PPI withdrawal considering NERD and RE patients had similar GERD symptom severity. There were some differences in terms of dietary habits, dietary and lifestyle triggers, and related quality of life between NERD and RE, and these results may provide evidence of different approaches toward the dietary modification of NERD and RE patients.

## 1. Introduction

Gastroesophageal reflux disease (GERD) is the condition in which the reflux of gastric contents into the esophagus results in symptoms and/or complications [1,2]. Typical symptoms include heartburn and regurgitation. The extra-esophageal symptoms present in about one-third GERD patients, including laryngeal, oropharyngeal, pulmonary, and sleep-related symptoms [3,4]. All these symptoms adversely affect health-related quality of life, especially when complications occur, such as esophageal ulceration, stricture, perforation, metaplasia, and adenocarcinoma. The overall pooled prevalence of GERD is about 14.0% and varied greatly according to region and country. The highest prevalence was in North America (19.6%), followed by Europe (14.1%), and Asia (12.9%) [5]. Over the past two decades, the prevalence of GERD in China has increased from 6.0% to 10.6% [6]. The incidence was approximately 5 and 7 per 1000 person years in the Western world and China, respectively [7,8]. GERD has brought substantial economic and health burdens to patients and society. The annual direct cost of GERD management in the USA is ranging from USD 9.3 to 12.1 billion, especially in patients with extra-esophageal symptom [7,9].

According to esophageal mucosa injury condition under endoscopy, GERD could be categorized into non-erosive reflux disease (NERD), reflux esophagitis (RE), and Barrett’s esophagus (BE). The most common subtype is NERD, which accounts for 60–70% of GERD patients, followed by RE (30%) and BE (6–8%) in Western counties [10]. The pathophysiology of GERD is multifactorial, including a poorly functioning anti-reflux barrier, impaired esophageal clearance and motility, decreased salivary production, delayed gastric emptying, and esophageal mucosal integrity and hypersensitivity (especially in NERD) [2,3,11].

The epidemiological studies of Western countries revealed GERD is associated with some dietary triggers, including increased consumption of high fat diet, sweets, chocolate, coffee and caffeine, spicy food, carbonated beverages, and acidic foods, such as citrus and tomatoes, salt, tobacco, and alcohol, while it is inversely associated with consumption of fruit and fiber [12,13,14]. Eating habits such as irregular meal pattern, large volume of meals, and eating meals just before bedtime may correlate with reflux symptom onset. Obesity is a strong risk factor for GERD [3,12,15]. It was noted there are some differences in the associated dietary and lifestyle risk factors between NERD and RE, which seem to have different pathophysiological mechanisms and clinical characteristics [11]. It is reported that female gender, younger age, low body mass index (BMI), non-smoking, absence of hiatal hernia, psychological distress, and severity of gastric atrophy were positively associated with NERD compared with RE in Japanese patients [16,17,18]. In Korean subjects, a higher dietary intake of beans, fruits, vegetables, seaweed, fish, milk, eggs, and drinking tea reduced NERD prevalence, but the dietary component had little effect on RE, while RE was associated with men, high BMI, alcohol, hiatal hernia, and total energy intake rather than dietary component [19,20]. Dietary and lifestyle modifications are first-line therapy for GERD patients, including avoidance of the trigger foods above, reducing or quitting tobacco/smoking, weight loss in overweight and obese patients, and elevating the head of the bed at nighttime [2].

Dietary habits and lifestyles differ between regions. There are various dietary cuisines, including food categories and cooking methods in China, which makes it difficult to identify the dietary risk factors of GERD. While GERD has increased over the past few decades, there are few studies on differences in diet and lifestyle triggers between NERD and RE in the Chinese population. Therefore, we aimed to investigate the diet and lifestyle triggers between RE and NERD in a large multicenter cross-sectional survey to understand the comprehensive relationship between the patient’s diets and lifestyles and reflux symptoms and to provide more lifestyle advices for GERD patients with similar dietary culture.

## 2. Materials and Methods

### 2.1. Study Design and Population

Consecutive adult patients with GERD aged 18–80 years were enrolled in this study from the gastroenterology clinics in four tertiary hospitals from November 2011 to October 2012. All patients had experienced typical reflux symptoms, i.e., mild heartburn and/or regurgitation symptoms, for at least 2 days per week or moderate/severe reflux symptoms for more than 1 day per week [1] during the previous 3 months and had upper endoscopy examinations in the previous month, which was used to classify GERD into NERD and RE as well as the severity of RE graded from A to D according to the Los Angeles classification [21]. Patients with secondary esophagitis, peptic ulcer, malignant cancer, and surgery of upper gastrointestinal tract were excluded from this study. All participants provided oral consent to participate before the enrollment.

The study was designed to consecutively enroll 100 patients with NERD and 100 patients with RE in each hospital.

The study was approved by the Ethics Committee of Peking Union Medical College Hospital (Project identification code I-23PJ1009).

### 2.2. Questionnaires and Data Collection

“Questionnaire of GERD and diets” consists of demographic data, GERD symptoms and extra-esophageal symptoms, GERD-related tests and previous management, alarm signs and comorbid diseases, psychological and sleeping status, dietary habits and lifestyle, dietary and other triggers for reflux symptoms, and so on. The typical GERD symptoms include heartburn, acid reflux, food regurgitation, and retrosternal chest pain. The severity of each symptom during the previous 3 months was graded as follows: 0 = no symptom, 1 = mild symptoms which can be felt when paying attention and no interference with normal activity or sleep; 2 = moderate symptoms between mild and severe and mild interference with normal daily activities or sleep; and 3 = severe symptoms and marked interference with normal daily activities or sleep. The frequency of each symptom was recorded as days per week and further scored as follows: 0 = none; 1 = <2 days per week; 2 = 2–4 days per week; 3 = >4 days per week [22]. The GERD symptom score was the product of severity and frequency. The extra-esophageal symptoms include swallow related pain or dysphagia, respiratory (chronic cough, asthma, short of breath, and snore), laryngopharyngeal (hoarseness, sensation of a lump in the throat, sore or burning throat), and oral symptoms (toothache, dental erosion). The extra-esophageal symptoms were recorded at the levels of severity mentioned above.

According to Chinese’s dietary peculiarities, we asked the following questions concerning diet habits and lifestyle in the questionnaire: the type of staple food, the proportion of staple foods and dishes, the amounts of vegetables/fruits and meat consumed, food preferences (listing eight kinds of common foods), eating habits (degree of satiety and eating speed), association of reflux symptoms and meals in time, and smoking and drinking. Among the factors that may trigger reflux symptoms, 25 dietary and 6 other factors (including lifestyle) were listed for patients to choose and answer with options “never triggered, occasionally, sometimes, and often or never experienced”.

The questionnaires also included questions about anxiety, depression, and sleeping disorders, such as “In the last 3 months, have you felt tense or wound up?” and “In the last 3 months, have you felt downhearted and low?”. If participants answered these questions with “often” (≥2 days a week) or “most of the time” or “always”, they were defined as having self-reported anxiety or depression in this study [8,23]. Sleeping disorders were defined as difficulty in falling asleep, early awakening, shallow sleep, sleep time < 6 h, or daytime drowsiness. The questionnaire was completed by well-trained investigators in face-to-face interviews. The coexisting functional gastrointestinal disease, including functional dyspepsia (FD), functional constipation (FC), irritable bowel syndrome (IBS), and psychological and sleep disorders were recorded, which were diagnosed according to the Rome III diagnostic criteria [23].

The quality of life was assessed by gastroesophageal reflux disease health-related quality of life (GERD-HRQL) and 36-item short-form health survey (SF-36). These two instruments were completed by patients according to the instruction provided, and the total scores were calculated as in the previous publications [24,25].

### 2.3. Statistical Analysis

All statistical analyses were conducted by SPSS 25 (IBM, Armonk, NY, USA). We used mean ± standard deviation for continuous variables with normal distribution, median (interquartile range) for those without normal distribution, and count with percentage for categorical variables. Univariate analyses were performed using parametric (Student’s *t*-test) or non-parametric methods (Mann–Whitney U test) for continuous variables, and chi-square test for categorical variables. NERD and RE patients were compared to assess their differences in demographic data, clinical symptoms, dietary habits, trigger factors, and management. *p*-Values are two-sided and considered significant when <0.05. Pearson’s test and Spearman’s test were performed to assess correlations between two quantitative variables with normal distribution and without normal distribution.

## 3. Results

### 3.1. Demographic Data

In total, 396 GERD patients were enrolled in this study, with an average age of 46.8 ± 12.7 years. There were 238 males (60.1%) and 158 females (39.9%). There were 203 NERD patients (51.3%) and 193 RE patients (48.7%). Among RE patients, 95 patients (49.2%) were classified as LA-A, and 76 (39.4%), 20 (10.4%), and 2 (1.0%) were classified as LA-B, LA-C, and LA-D respectively. Male patients were more likely to present with RE than female patients, and there were no significant differences in age, BMI, and other demographic characteristics (Table 1).

### 3.2. Characteristics of Reflux Symptoms

There were no significant differences in general GERD symptom scores and the scores of heartburn, acid reflux, food regurgitation, and retrosternal chest pain between NERD and RE patients (Table 2).

There was no significant difference in extra-esophageal symptom score, including swallowing-related symptoms, respiratory symptoms, and laryngopharyngeal symptoms. The prevalence of oral symptoms of NERD patients was higher than for RE patients (Table 2).

In addition to GERD symptoms and extra-esophageal symptoms, we found NERD patients were more likely to coexist with FD and FC than RE patients. NERD patients had higher prevalence of self-reported anxiety and depression than RE patients, and more NERD patients thought that their reflux symptoms were related to emotion than RE patients (Table 2). NERD patients had a higher prevalence of sleep disorders but without significant difference compared with RE patients.

We collected the data for pH or pH-impedance monitoring in 26 (12.8%) NERD patients and 15 (7.8%) RE patients, and we collected the data for esophageal manometry in 19 (9.4%) NERD patients and 12 (6.2%) RE patients while they were enrolled in this survey. We did not further analyze the association of dietary and lifestyle triggers and esophageal reflux and motility parameters in NERD and RE patients.

### 3.3. Characteristics of GERD Management

We summarized GERD management during the whole disease. Sixty-five (16.4%) patients used medications long term to treat GERD. Proton-pump inhibitors (PPIs) were mostly used by 225 (56.8%) patients and the effective rate was 50.2%. The relapse rate after PPI withdrawal was as high as 88.0%. There were no significant differences in patients who mostly used PPIs, H_2_-receptor antagonists, or antacids and the effective rate and relapse rate after drug withdrawal between NERD and RE patients. In addition to medications, 93 (23.5%) patients raised the head of the bed, 99 (25.0%) patients stopped smoking and drinking, 96 (24.2%) patients chose a low-lipid and low-sugar diet, 174 (43.9%) patients avoided overeating to relieve symptoms, and 44.4% and 45.7% of NERD patients reported the effectiveness from a low-lipid/low-sugar diet and avoiding overeating respectively. There were no significant differences in the proportions of the above management strategies and effective rates between NERD and RE patients (Table 3).

### 3.4. Dietary Habits

We compared dietary habits between NERD patients and RE patients. The RE group had a higher proportion of overeating than NERD patients (26.4% vs. 17.2%, *p* = 0.027). There were no significant differences in fast eating, degree of satiety, dominant food type, dish style, and consumption of fruits or meat between the two groups. The rates of patients who often consumed spicy food, greasy food, sweet food, sticky food, cold food, carbonated beverages, coffee, and strong tea had no significant difference between the two groups. In the RE group, more patients had smoking and drinking habits than did NERD patients (Table 4).

### 3.5. Triggers of GERD Symptoms

**Figure 1:**
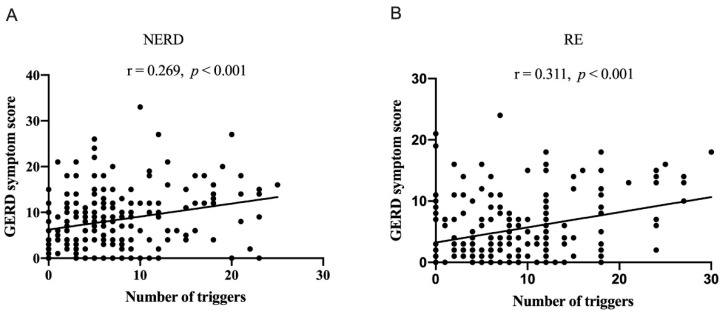
Correlation analysis of the number of triggers with GERD symptom score in NERD (**A**) and RE (**B**) patients.

We compared dietary and other lifestyle triggers that sometimes and often induced GERD symptoms between NERD patients and RE patients (Table 5). In the NERD group, more patients reported fruits, dairy products, yogurt, bean products, uncooked food, cold food, midnight snacks, sweet food, cold weather, and emotional disturbance sometimes and often induced GERD symptoms than did RE patients. The mean number of triggers was higher in NERD patients than in RE (5 [8] vs. 4 [6], *p* = 0.007). In all of the GERD patients, 346 (87.4%) patients had at least one trigger and 274 (69.2%), 204 (51.5%), 90 (22.7%), and 13 (3.3%) patients had at least 3, 5, 10, and 20 triggers, respectively. The percentages of patients who had at least 3, 5, and 20 triggers in the NERD group were higher than the RE group (74.9% vs. 63.2%, *p* = 0.012; 59.6% vs. 43.0%, *p* = 0.001; and 5.4% vs. 1.0%, *p* = 0.014, respectively). The number of triggers was positively correlated to the GERD symptom score in both NERD and RE patients (Figure 1).

### 3.6. Diet Modification Based on Triggers

We analyzed whether patients would avoid their reflux-symptom-trigger foods. We found in patients with rice as a trigger, a higher percentage of RE patients still ate rice compared with NERD patients. While in patients with cooked wheaten food as a trigger, a higher percentage of NERD patients still ate cooked wheaten food compared with RE patients. In patients with fruits, spicy food, greasy food, uncooked food, cold food, carbonated beverages, coffee, strong tea, sweet food, and sticky food as triggers, a considerable number of patients still often ate these foods, especially strong tea, spicy food, sweet food, and cold food (Table 6). In total, 74.0% (214/289) of patients still often consumed the triggering foods, even those foods that sometimes and often induced their reflux symptoms, and RE patients had higher percentage than NERD patients (82.1% vs. 66.4%, *p* = 0.002), while there were no significant differences in the percentage of patients who still often ate any type of triggering food between NERD and RE except the staple foods mentioned above.

### 3.7. GERD-HRQL Score and SF-36 Total Score

**Figure 2:**
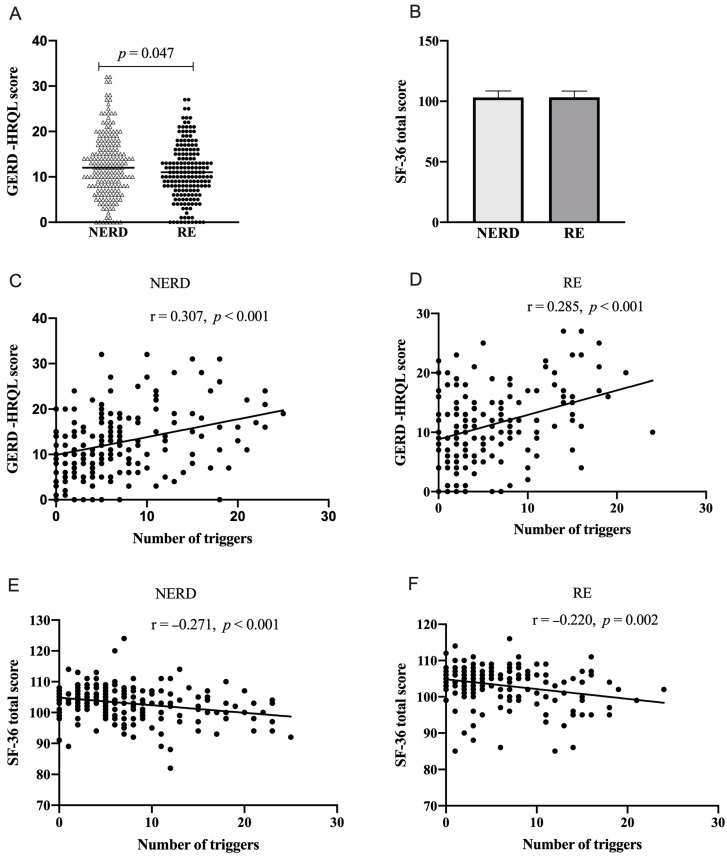
Comparison of GERD-HRQL and SF-36 total score and correlation analysis of the number of triggers with GERD-HRQL and SF-36 total score in NERD and RE patients. (**A**) comparison of GERD-HRQL between NERD and RE patients. (**B**) Comparison of SF-36 total score between NERD and RE patients. (**C**,**D**) Correlation analysis of the number of triggers with GERD-HRQL in NERD and RE patients. (**E**,**F**) Correlation analysis of the number of triggers with SF-36 total score in NERD and RE patients.

NERD patients had higher GERD-HRQL scores than RE patients (Figure 2A), indicating NERD patients had worse GERD-related quality of life. There was no significant difference in SF-36 total score between NERD patients and RE patients (Figure 2B). The number of triggers was positively correlated to GERD-HRQL score (Figure 2C,D) and negatively correlated to SF-36 total score (Figure 2E,F) in both NERD and RE patients.

## 4. Discussion

This is a large-scale, multicenter, cross-sectional survey on dietary and lifestyle triggers of NERD and RE patients in China. We found there were no significant differences in GERD symptom scores between NERD and RE, while RE patients had a higher male proportion, smoking/drinking rate, and overeating rate than NERD patients. In total, 87.4% GERD patients had at least one trigger for their reflux symptoms, and NERD patients had more triggers than RE. The number of triggers was positively correlated to GERD symptom scores and GERD-HRQL scores; meanwhile, they were negatively correlated to SF-36 scores. Of those who took dietary modifications, 44.4% and 45.7% of NERD patients reported the effectiveness from low-lipid/low-sugar food and avoiding overeating, respectively, while as high as 82.1% RE patients often consumed triggering foods, even those foods that sometimes and often induced their reflux symptoms.

In our result, we found there was no significant difference in major GERD symptoms and most extra-esophageal symptoms between NERD and RE patients, which was consistent with a previous study [26]. The NERD consensus indicated symptom severity does not allow confident differentiation between NERD and erosive reflux disease [11]. There are many similarities between NERD and RE in the perspective of pathophysiological mechanisms. RE is characterized by excessive esophageal acid exposure and esophageal mucosa injury under endoscopy. Generally, the number of acid reflux episodes and the delay of acid bolus clearance cause excessive acid exposure [27]. RE seemed to have more obvious esophageal motor dysfunctions and injury of the mucosa barrier, and the pathophysiology of NERD was poorly understood. The mechanism of esophageal mucosa injury in RE patients compared to NERD patients with similar symptom severity was not completely clear. In this study, the self-reported effective rate of PPI was about 50%, but the relapse rate after PPI withdrawal was over 80%, which indicated there are some lifestyle and dietary aspects combined with dysmotility of the esophagus to enhance the reflux persistence.

Considering the potential side effects of long-term pharmacotherapy, lifestyle modifications are increasing in popularity. Although dietary manipulation is commonly employed in clinical settings, data are conflicting for definitive recommendations. Various lifestyle factors, such as smoking, alcohol intake, poor dietary habits (large volume of meals, lying down after a meal, meals just before bedtime) and lack of regular physical activity have been reported to be risk factors of GERD occurrence [2]. Studies indicated high-fat and high-caloric-load food; fried, sour, and spicy food; chocolate; and coffee/tea as triggers for GERD symptoms [15,18,28,29]. High-fat meals could significantly increase postprandial esophageal acid exposure in both RE and NERD [30]. Some diets were related to a proposed mechanism of GERD symptoms, coffee, alcohol, chocolate, mint and fats were related to reduction in lower esophageal sphincter (LES) tone, and late-night meals were related to increased gastric acid production [31], but few studies document the benefits of avoiding these foods. Rare data exist regarding the association between diet in RE and NERD, especially in China, where foods and cooking methods are very rich and diversified, which makes it difficult to determine the dietary risk factors for reflux symptoms in clinical practice. In this study, we set as many questions as possible and easy-to-answer options for participants, including the eating habits and triggering factors according to the dietary particularities of the Chinese, and aimed to understand the comprehensive relationship between the patients’ diets and lifestyles and reflux symptoms.

With regard to ordinary dietary habits, we found more RE patients reported overeating, smoking, and drinking than NERD patients, and RE patients had a tendency to report higher percentages of fast eating than NERD patients. We found that 87.4% GERD patients had at least one trigger for their reflux symptoms, and NERD patients had more triggers than RE patients. GERD patients seemed to have similar most-common triggers; in NERD patients, the top five triggers were cold weather, emotional disturbance, weather change, spicy food, and drinking, and in RE patients, the top five triggers were drinking, cold weather, spicy food, weather change, and emotional disturbance. Thus, in clinical settings, we recommend GERD patients to avoid the five top triggers. More NERD patients reported fruits, dairy products, yogurt, bean products, uncooked food, cold food, midnight snacks, sweet food, cold weather, and emotional disturbance sometimes and often induced GERD symptoms than RE patients. The percentages of patients who had at least 3, 5, and 20 triggers in the NERD group were higher than the RE group. We assumed that NERD patients were more concerned or sensitive to dietary and lifestyle triggers than RE patients. Indeed, we also found NERD patients were more likely to coexist with FD, FC, and psychological disorders, indicating the important role of visceral hypersensitivity and psychological factors in the pathogenesis and perception of symptoms of NERD [11,32] as well as reporting more triggers for their reflux symptoms.

It is disappointing that 74.0% of GERD patients still often consumed the triggering foods, even those foods that sometimes and often induced their reflux symptoms, and RE patients had a higher percentage than did NERD patients (82.1% vs. 66.4%, *p* = 0.002). Considering that NERD and RE patients had similar GERD symptom severity with the latter group presenting with esophageal mucosal injury, we supposed that dietary habits like higher percentage of overeating, smoking, and drinking and more patients still often consuming the triggering foods were closely related to more severe acid exposure in RE patients than in NERD patients.

NERD patients had worse GERD-related quality of life than RE patients, while both groups have comparable GERD symptom scores and prevalence of extra-esophageal symptoms (except of oral symptom). The number of triggers was positively correlated to GERD-HRQL score and negatively correlated to SF-36 total score. We speculated that NERD patients with more dietary triggers might be more worried about their types of foods and excessive diet avoidance, and anxiety adversely affected their quality of life. Therefore, identification and effective management of the adverse effect of dietary and lifestyle triggers to reflux symptoms and quality of life was significant for NERD patients.

Management of GERD requires a multifaceted approach; PPI and other acid suppression therapy were first-line treatments. We observed the relatively low effective rate of PPI and high relapse rate after PPI withdrawal. A study showed the relapse rate of 4 weeks and 12 weeks after PPI treatment suspension was 48.64% and 17.57%, and they found citrus fruits (OR = 14.76) were independent risk factors of GERD relapse [33]. A Japanese study showed hiatus hernia and past severe erosive GERD (grade C or D) were risk factors for RE relapse during long-term maintenance treatment with PPIs [34]. Diet modification includes manipulation of meal size, avoidance of late-night meals and bedtime snacks, tobacco and alcohol cessation, and cessation of foods that potentially aggravate reflux symptoms, such as coffee, chocolate, carbonated beverages, spicy foods, acidic foods, such as citrus and tomatoes, and foods with high fat content were recommended in the literature [31,35]. Supporting data for these suggestions were limited and variable. According to our study, we recommend trying to avoid cold weather, emotional disturbance, weather change, spicy food, and drinking in all GERD patients. Additionally, we advise GERD patients to avoid smoking, drinking, and overeating, especially in RE patients. Also, we recommend properly avoiding the intake of fruits, dairy products, yogurt, bean products, uncooked food, and cold food, especially in NERD patients. We hope that these recommendations will be useful to patients with GERD who have similar food habits and dietary cultures. More importantly, good education on dietary and lifestyle modification are needed for GERD patients, especially emphasizing the avoidance of the intake of already known symptom-triggering foods, which is basic and important for permanent management of GERD. Further well-designed randomized, controlled trials are still needed to study the effects of dietary modification on GERD management.

There were several limitations of our study. Firstly, this study was retrospective with a food survey that spans three months prior to the survey and therefore requires a mnemonic effort and an important involvement of the subjects. Secondly, less than 20% NERD and RE patients had pH or pH-impedance monitoring tests, and less than 10% NERD and RE patients had esophageal manometry data. NERD was diagnosed with typical symptoms and self-reported response to PPIs, not rigorously distinguished from acid reflux, non-acid reflux, or functional heartburn, and we did not explore the relationship among GERD symptoms, dietary and lifestyle triggers, and pathophysiological mechanisms. Thirdly, we only collected data of GERD management during the whole disease, including PPI use and response, but we did not perform PPI tests after patients’ enrollment. Finally, we did not observe the real effective rate of dietary modifications. In addition, the healthcare systems in China permmit for both referral and non-refereral patients to have a registered appointment in the tertiary hospitals when they would like to seek more professional consultation; we hope our results could be referred to those patients in parimary care.

## 5. Conclusions

GERD is a common gastrointestinal disease which is closely related to diet habits and lifestyles. Of the 87.4% GERD patients who had at least one trigger for their reflux symptoms, the number of triggers had close association to GERD symptoms and quality of life. Both NERD and RE patients had high relapse rates after PPI withdrawal, which could be related to dietary triggers. NERD patients had more dietary triggers and psychological factors than RE patients, indicating the underlying pathophysiology of visceral hypersensitivity, while more RE patients had poor dietary habits and often consumed the triggering foods indicating the potential effect on prolonged esophageal acid exposure. It is important to educate those GERD patients to modify their poor dietary habits and avoid already known symptom triggers based on individual triggers, especially for RE patients in the long-term course of therapy.

## Figures and Tables

**Table 1:** Demographic data of RE and NERD patients.

Variable	NERD (*n* = 203)	RE (*n* = 193)	*p*-Value
Age (years)	45.1 ± 12.8	48.5 ± 12.3	0.729
Gender, *n* of male (%)	94 (46.3)	144 (74.6)	<0.001 *
BMI (kg/m^2^)	22.8 ± 3.1	23.7 ± 3.0	0.472
Education level, *n* of college and above (%)	49 (24.2)	48 (24.8)	0.865
Family economic status, *n* of well-off and above (%)	94 (46.3)	72 (37.3)	0.070
Marriage status, *n* of married (%)	179(88.2)	179 (92.7)	0.123
Labor intensity, *n* of severe (%)	15 (7.4)	16 (8.3)	0.739

Data presented as mean ± standard deviation or number (%). Student’s *t*-test and chi-square test. * *p*-value < 0.05.

**Table 2:** Characteristics of clinical manifestations of RE and NERD patients.

Variable	NERD (*n* = 203)	RE (*n* = 193)	*p*-Value
**GERD symptom score**	7 (8)	8 (8)	0.277
Heartburn	3 (6)	3 (6)	0.460
Acid reflux	2 (6)	3 (5.8)	0.078
Food regurgitation	0 (0)	0 (0)	0.608
Retrosternal chest pain	0 (2)	0 (2)	0.723
**Extra-esophageal symptoms**			
Swallowing-related symptoms, *n* (%)	39 (19.2)	25 (13.0)	0.091
Respiratory symptoms, *n* (%)	120 (59.1)	122 (63.2)	0.403
Laryngopharyngeal symptoms, *n* (%)	119 (58.6)	98 (50.7)	0.117
Oral symptoms, *n* (%)	54 (26.6)	25 (13.0)	0.001 *
Coexisting functional dyspepsia, *n* (%)	63 (31.0)	32 (16.6)	0.001 *
Coexisting functional constipation, *n* (%)	19 (9.4)	8 (4.1)	0.040 *
Coexisting irritable bowel syndrome, *n* (%)	7 (3.4)	2 (1.0)	0.107
Feelings of anxiety, ^#^ *n* (%)	80 (39.4)	42 (21.8)	<0.001 *
Feelings of depression, ^#^ *n* (%)	64 (31.5)	40 (20.7)	0.015 *
GERD symptoms related to emotion, *n* (%)	50 (24.6)	24 (12.4)	0.002 *
Sleep disorder, *n* (%)	77 (37.9)	57 (29.5)	0.078
GERD symptoms related to sleep disorder, *n* (%)	60 (29.6)	40 (20.7)	0.083

Data presented as median (interquartile range) or number (%). Mann–Whitney test and chi-square test. * *p* < 0.05. ^#^ Patients with sometimes, often, and always had feelings of anxiety or depression.

**Table 3:** Management of RE and NERD patients.

Variable	NERD (*n* = 203)	RE (*n* = 193)	*p*-Value
Long-term use of medications	36 (17.7)	29 (15.0)	0.467
Proton-pump inhibitor was mostly used	125 (61.6)	100 (51.8)	0.050
Effective rate	63 (50.4)	50 (50.0)	0.952
Relapse after withdrawal	111 (88.8)	87 (87.0)	0.680
H_2_-receptor antagonist was mostly used	27 (13.3)	24 (12.4)	0.797
Effective rate	12 (44.4)	10 (41.7)	0.842
Relapse after withdrawal	22 (81.5)	19(79.2)	0.835
Antacid was mostly used	29 (14.3)	19 (9.8)	0.176
Effective rate	11 (37.9)	4 (21.1)	0.217
Relapse after withdrawal	26 (89.5)	16 (84.2)	0.577
Raise the head of bed	60 (29.6)	33 (17.1)	0.354
Effective rate	20 (33.3)	11 (33.3)	1.000
Stop smoking and drinking	45 (22.2)	54 (28.0)	0.182
Effective rate	14 (31.1)	19 (35.2)	0.669
Low-lipid and low-sugar diet	54 (26.6)	42 (21.8)	0.261
Effective rate	24 (44.4)	12 (28.6)	0.111
Avoid overeating	92 (45.3)	82 (42.5)	0.570
Effective rate	42 (45.7)	27 (33.0)	0.087

Data presented as number (%). Chi-square test.

**Table 4:** Comparison of dietary habits between RE and NERD patients.

Variable	NERD (*n* = 203)	RE (*n* = 193)	*p*-Value
Overeating, *n* (%)	35 (17.2)	51 (26.4)	0.027 *
Fast eating, *n* (%)	77 (37.9)	91 (47.2)	0.064
Degree of satiety (0–10)	8.9 ± 6.0	8.5 ± 1.2	0.936
**Dominant food**			0.163
Staple food, *n* (%)	86 (42.4)	64 (33.2)	
Dish food, *n* (%)	15 (7.4)	18 (9.3)	
Both equivalent, *n* (%)	102 (50.2)	111 (57.5)	
**Dish styles**			0.055
More vegetables, *n* (%)	89 (43.8)	63 (32.6)	
More meat, *n* (%)	17 (8.4)	24 (12.4)	
Both equivalent, *n* (%)	97 (47.8)	106 (54.9)	
**Consumption of fruits**			0.319
Seldom, *n* (%)	51 (25.1)	45 (23.3)	
Moderate, *n* (%)	137 (67.5)	140 (72.5)	
Much, *n* (%)	15 (7.4)	8 (4.1)	
**Consumption of meat**			0.087
Seldom, *n* (%)	66 (32.5)	44 (22.8)	
Moderate, *n* (%)	121 (59.6)	138 (71.5)	
Much, *n* (%)	15 (7.4)	11 (5.7)	
**Often consumption of foods**			
Spicy, *n* (%)	33 (16.3)	35 (18.1)	0.145
Greasy, *n* (%)	25 (12.3)	28 (14.5)	0.386
Sweet food, *n* (%)	22 (10.8)	16 (8.3)	0.390
Sticky food, *n* (%)	8 (3.9)	7 (3.6)	0.870
Cold food, *n* (%)	19 (9.4)	9 (4.7)	0.068
Acid drink, *n* (%)	6 (3.0)	5 (2.6)	0.238
Coffee, *n* (%)	2 (1.0)	6 (3.1)	0.280
Strong tea, *n* (%)	1 (0.5)	1 (0.5)	0.549
Smoking, *n* (%)	41 (20.2)	67 (34.8)	0.001 *
Drinking, *n* (%)	42 (20.7)	74 (38.3)	<0.001 *

Data presented as mean ± standard deviation or number (%). Student’s *t*-test and chi-square test. * *p* < 0.05.

**Table 5:** Comparison of triggers that sometimes and often induced GERD symptoms between RE and NERD patients.

Triggers	NERD (*n* = 203)	RE (*n* = 193)	*p*-Value
Rice	31/198 (15.7)	26/192 (13.5)	0.554
Cooked wheaten food	30/202 (14.9)	33/193 (17.1)	0.542
Wheat bran	12/139 (8.6)	6/136 (4.4)	0.157
Fruits	43/195 (22.1)	15/189 (7.9)	<0.001 *
Shallot or fragrant-flowered garlic	73/191 (38.2)	58/187 (31.0)	0.141
Dairy products	30/179 (16.8)	15/178 (8.4)	0.018 *
Yogurt	29/174 (16.7)	14/172 (8.1)	0.016 *
Bean products	29/197 (16.2)	5/190 (2.6)	<0.001 *
Meat	34/197 (19.0)	35/189 (18.5)	0.747
Salted food	37/169 (21.9)	32/169 (18.9)	0.500
Spicy food	94/161 (58.4)	95/171 (55.6)	0.603
Greasy food	61/178 (34.3)	65/183 (35.5)	0.803
Uncooked food	51/161 (31.8)	29/175 (16.6)	0.001 *
Cold food	58/161 (36.0)	30/175 (17.1)	<0.001 *
Carbonated beverages	27/126 (21.4)	32/136 (23.5)	0.684
Coffee	14/66 (21.2)	15/73 (20.5)	0.923
Strong tea	20/91 (22.0)	26/105 (24.8)	0.646
Drinking	44/96 (45.8)	62/108 (57.4)	0.099
Dining out	31/155 (20.0)	22/155 (14.2)	0.175
On business	22/120 (18.3)	14/128 (10.9)	0.098
Overeating	80/174 (46.0)	71/168 (42.3)	0.489
Diet food	4/45 (8.9)	2/34 (5.9)	0.617
Midnight snacks	35/117 (30.0)	21/121 (17.4)	0.022 *
Sweet food	76/177 (43.0)	48/177 (27.1)	0.002 *
Sticky food	37/152 (24.3)	28/161 (17.4)	0.130
Cold weather	71/100 (71.0)	40/71 (56.3)	0.048 *
Weather change	54/88 (61.4)	28/59 (47.5)	0.096
Emotional disturbance	77/119 (64.7)	36/84 (42.9)	0.002 *
Sleep disorder	43/99 (43.4)	26/73 (35.6)	0.301
Bedtime meal	42/124 (33.9)	31/109 (28.4)	0.373
Postprandial bending	41/105 (39.0)	35/94 (37.3)	0.793

Data presented as number (%); the number of denominators refers to the number of patients who had this experience. Chi-square test. * *p* < 0.05.

**Table 6:** Comparison of patients who still often consumed foods which sometimes and often induced GERD symptoms.

Variables	NERD (*n* = 203)	RE (*n* = 193)	*p*-Value
Rice as staple food	5/31 (16.1%)	12/26 (46.2%)	0.014 *
Cooked wheaten food as staple food	21/30 (70.0%)	14/33 (42.4%)	0.028 *
Fruits (often)	4/43 (9.3%)	1/15 (6.7%)	0.754
Spicy food (often)	23/94 (24.5%)	26/95 (27.4%)	0.649
Greasy food (often)	11/61 (18.0%)	15/65 (23.1%)	0.484
Uncooked food (often)	10/51 (19.6%)	2/29 (6.9%)	0.126
Cold food (often)	12/58 (20.7%)	2/30 (6.7%)	0.088
Carbonated beverages (often)	2/27 (7.4%)	3/32 (9.4%)	0.787
Coffee (often)	2/14 (14.3%)	1/15 (6.7%)	0.501
Strong tea (often)	6/20 (30.0%)	8/26 (30.8%)	0.955
Drinking	30/44 (68.2%)	51/62 (82.3%)	0.093
Sweet food (often)	18/76 (23.7%)	11/48 (22.9%)	0.922
Sticky food (often)	6/37 (16.2%)	4/28 (14.3%)	0.879

Data presented as number (%). Chi-square test. * *p* < 0.05.

## Data Availability

The datasets used and/or analyzed during the current study are available from the corresponding author on reasonable request.

## Abbreviations and Acronyms

GERD	gastroesophageal reflux disease
NERD	non-erosive reflux disease
RE	reflux esophagitis
BE	Barrett’s esophagus
BMI	body mass index
FD	functional dyspepsia
FC	functional constipation
IBS	irritable bowel syndrome
GERD-HRQL	gastroesophageal reflux disease health-related quality of life
SF-36	36-item short-form health survey
PPI	proton pump inhibitor
